# Molecular detection and identification of *Leishmania* infection in naturally infected sand flies in a focus of cutaneous leishmaniasis in northern Morocco

**DOI:** 10.1186/1756-3305-7-305

**Published:** 2014-07-02

**Authors:** Nargys Es-Sette, Malika Ajaoud, Abderrahman Laamrani-Idrissi, Fouad Mellouki, Meryem Lemrani

**Affiliations:** 1 Laboratoire de Parasitologie et Maladies Vectorielles, Institut Pasteur du Maroc, 1 Place Louis Pasteur, 20360 Casablanca, Morocco; 2 Laboratoire de chimie Bioorganique et analytique, URAC C22, faculté des sciences et techniques, Université Hassan II Mohammedia-Casablanca, Mohammedia, Morocco; 3 Service de Parasitologie, DELM, Ministère de la Santé, Rabat, Morocco

**Keywords:** *P. sergenti*, *P. longicuspis*, *L. tropica*, *L. infantum*, Cutaneous leishmaniasis, Blood meal, Morocco

## Abstract

**Background:**

Cutaneous leishmaniasis is an infectious disease caused by various species of the flagellate protozoan *Leishmania*. During the past 20 years, cutaneous leishmaniasis has emerged as a major public health threat in Morocco. The main objective of this study was to study the occurrence of *Leishmania* infection in vectors and to identify sand fly blood meal sources in an endemic locality of cutaneous leishmaniasis within Sefrou province, where the vectors of leishmaniasis were still unknown.

**Methods:**

2650 sand flies were collected using CDC miniature light traps and identified morphologically. The identified sand flies were tested for *Leishmania* infection by nested PCR. The source of blood meal of 10 freshly engorged females: 6 *Phlebotomus longicuspis* and 4 *Phlebotomus sergenti,* was determined using the *Cyt b* sequence.

**Results:**

The collected sand flies consisted of 10 species, seven of which belonged to the genus *Phlebotomus* and three to the genus *Sergentomyia*. The most abundant species was *P. longicuspis*, accounting for 72% of the total sand flies collected. In females of three *P. longicuspis* and four *P. sergenti*, *Leishmania infantum* and *Leishmania tropica* DNA was detected, respectively.

The source of blood meal of engorged females showed that all sand flies tested fed on humans.

**Conclusions:**

We report for the first time the natural infection of *P. longicuspis* with *L. infantum* in Morocco. The high frequency of this species in this region, in addition to its anthropophilic character make *P. longicuspis* the putative vector of *L. infantum* in this cutaneous leishmaniasis focus where *L. tropica* is confirmed as the causative agent of the disease and *P. sergenti* as its vector. The presence of *L. infantum*, and its presumed vector in this area, makes this a site of high risk of visceral leishmaniasis, mostly because of the proximity of a focus of human and canine visceral leishmaniasis.

## Background

Morocco lies in the Mediterranean region, where two clinico-epidemiological forms of leishmaniasis are endemic: visceral leishmaniasis (VL) and cutaneous leishmaniasis (CL). There were 9,000 CL cases reported in 2010 compared to 3,361 in 2006 and 655 in 1998, showing a clear tendency for an increase over the last decade [1]. Three species of *Leishmania* cause CL: *L. major*, *L. tropica* and less frequently *L. infantum*. Zoonotic CL caused by *L. major* is endemic especially in the southern slopes of the Atlas Mountains where its unique vector, *P. papatasi* and its reservoir host, *Meriones shawi*, are prevalent [2]. Anthroponotic CL due to *L. tropica* is endemic in arid and semi-arid regions in the center and mainly on the north western slopes of the Atlas Mountains. Its proven vector is *P. sergenti*[3].

Cutaneous leishmaniasis due to *L. infantum* occurs sporadically in the north of Morocco as reported by many authors [4-6]. *L. infantum* is also responsible for widespread visceral leishmaniasis in the North-Eastern slope of the Rif Mountains, the most active area of this form of the disease. The dog is the main domestic reservoir of the species [7]. Among about 20 sand fly species that play a significant role in the transmission of *Leishmania* parasites in the Mediterranean basin, members of the subgenus *Larroussius* represent the most important vectors of *L. infantum*[8-10]. In Morocco *P. perniciosus* and *P. longicuspis* were suspected as vectors of *L. infantum* based solely on their abundance in active foci of leishmaniasis, however, one specimen of *P. ariasi* caught inside a house in a focus of visceral leishmaniasis in northern Morocco was found to be naturally infected with promastigotes identified as *L. infantum* by immunofluorescence with monoclonal antibodies [11].

In northern Morocco, Sefrou province has been established as a focus of cutaneous and visceral leishmaniasis. According to the local health centers, sporadic human cases continue to occur every year in several localities of this province. The first CL cases appeared in 1997, the epidemiological situation remained stable until the year 2000. From 2000 to 2011, an average of 67 CL cases per year was recorded [1].

In 2008, the Moroccan Ministry of Health reported 9 VL cases and 64 CL cases spread over several sites belonging to Sefrou province, the causative agents of CL and VL were identified as *L. tropica*[12] and *L. infantum*, respectively [7,13,14]. Despite the large number of foci of CL and VL in this province, the vectors have never been identified with certainty.

The incrimination of a sand fly species as being the vector of *Leishmania* species is complicated; Killick –Kendrick [9] suggested the following criteria for incrimination of a vector sand fly: anthropophilic behavior and common infection with the same *Leishmania* parasite as that found in man in the same place. Further evidence such as the demonstration that the fly feeds regularly on the reservoir host, concordance in the distribution of the fly and the disease in man, proof that the parasite develops in infected flies and the experimental transmission of the parasite by the bite of the fly can reinforce the incrimination.

The classical method of *Leishmania* detection requires the dissection of freshly caught individual sand flies and culture of parasites found in the sand fly gut [15,16], but this technique is time-consuming, needs dissecting expertise and a large number of specimens, since the *Leishmania* infection rate in sand flies is usually very low even in highly endemic areas [17]. In recent years, molecular methods are increasingly employed in epidemiological studies to detect infection and to characterize *Leishmania* parasites in hosts and vectors; they are highly sensitive in the detection of *Leishmania* spp*.* infections in phlebotomines [18,19]. This sensitivity increases the understanding of vector competence and leishmaniasis epidemiology [18,19]. Accurate and sensitive diagnostic and identification procedures are also required to distinguish *Leishmania* species whose geographic distribution can overlap, which is crucial for appropriate public health control measures.

Hence, the objective of this work was to identify the species of phlebotomine sand flies involved in an endemic locality of cutaneous leishmaniasis within Sefrou province, where the vectors of leishmaniasis were still unknown. Molecular tools were used for the first time in the identification of the host blood feeding preferences of sand flies, together with the detection of parasitic infection within sand flies.

## Methods

### Study sites and collection of sand flies

**Figure 1:**
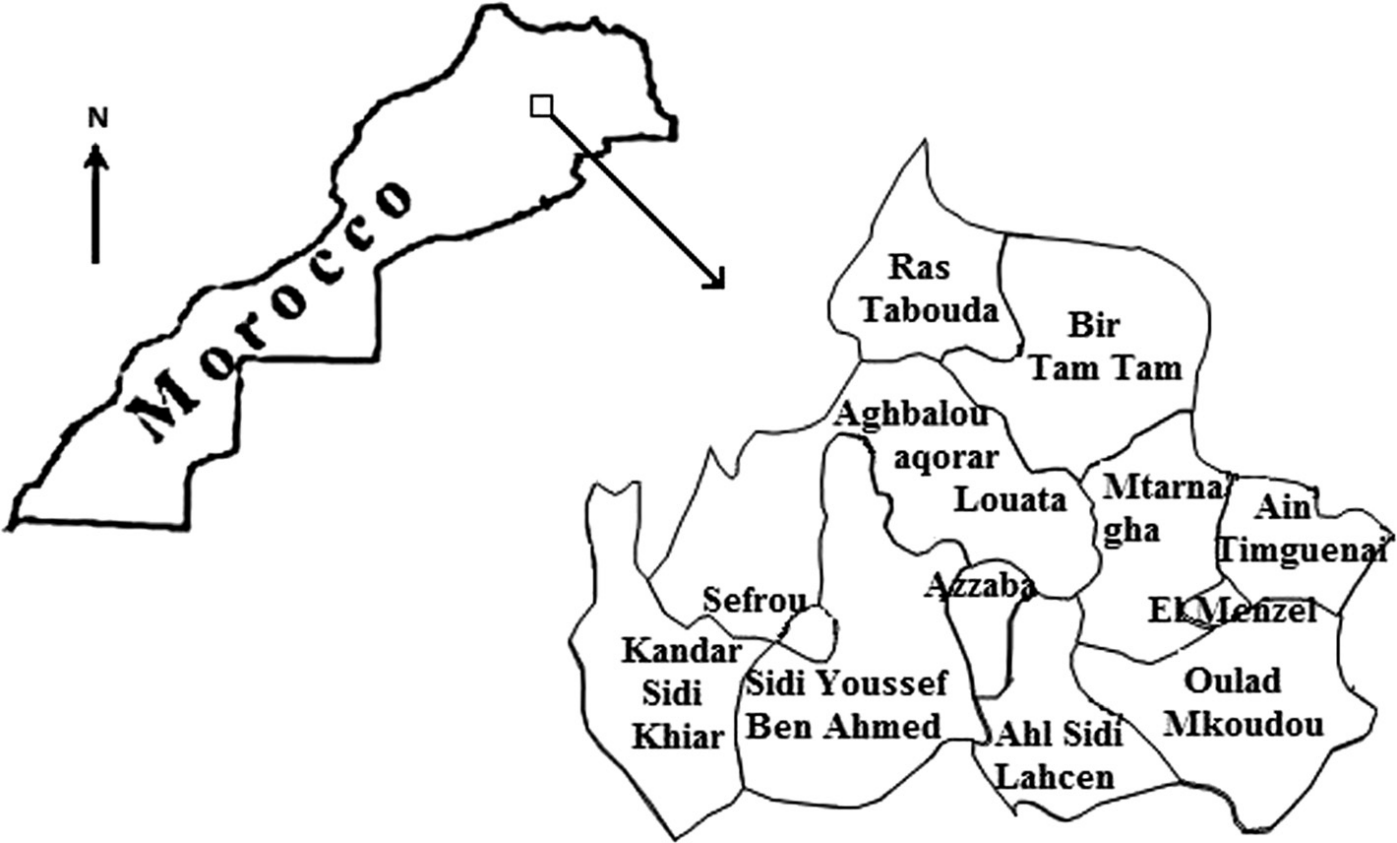
Map of Morocco showing Sefrou province and the Louata locality, the site of the survey.

Sand fly sampling was carried out from June-August 2008 (during the period of peak sand fly activity) in Louata, a locality situated in Sefrou province in northern Morocco (Figure 1). It is located in the north-west of the middle Atlas Mountain. Louata supports a semi-arid climate with mean annual rainfall of about 450 mm. The average elevation of Louata is 589 meters. Sand flies were collected by CDC light traps placed in or near houses or animal housing facilities. Traps were suspended so that the attracting light source was approximately 1.5 meters above the ground and were set in the late afternoon with collection occurring the following morning. The sand flies were then placed in 1.5 ml Eppendorf tubes and preserved at -80°C. Phlebotomine specimens of both genders were identified by their morphological characteristics. Each sand fly was dissected under a binocular microscope, on a sterilized microscopic slide using sterile steel entomological needles. The head and genitalia of each sand fly were mounted under a cover slip in Marc-André solution for morphological identification at the species level according to morphological keys described by the Moroccan Health Ministry [20]. After dissection, the abdomen and the thorax of each female specimen were transferred to sterile 1.5 ml Eppendorf tube. They were subsequently grouped into 55 pools according to date, collection site and species, with up to 30 specimens in each pool and were stored at -20°C. The preserved pools were processed for DNA extraction. For each female, it was determined whether it was engorged or unfed.

### Detection of *Leishmania* spp. in female sand flies

For each pool, DNA extraction was performed by the phenol-chloroform method [21]. DNA was purified by Qiagen kit according to the instructions of the manufacturer. Female sand flies were screened for *Leishmania* infection by nested PCR. Positive PCR were followed by direct sequencing.

### Nested PCR for amplifying the ITS-5.8S rDNA gene of *Leishmania* species

Each PCR was carried out in two separate tubes [22,23]. The first-stage PCR used the forward primer IR1 (5’ GCTGTAGGTGAACCTGCAGCAGCTGGATCATT 3’, at the 3’ end of the SSU rRNA gene) with the reverse primer IR2 (5’ GCGGGTAGTCCTGCCAAACACTCAGGTCTG 3’, at the 5’ end of the large subunit rRNA gene).

The ITS1-5.8S rDNA gene was amplified using the nested forward primer ITS1F (5’ GCAGCTGGATCATTTTCC 3’; overlapping the 3’ end of the SSU rRNA gene and ITS1) with the nested reverse primer ITS2R4 (5’ ATATGCAGAAGAGAGGAGGC 3’; at the 5’ end of ITS2) [23].

The first amplification reaction totaled 20 μl, containing 1xTaq polymerase buffer B (Invitrogen), 1.5 mM MgCl_2_, 60 μM each dNTP, 1 μM primer IR1, 1 μM primer IR2, 1 unit Taq polymerase (Invitrogen). The mixture was incubated in a thermocycler involving an initial denaturation at 94°C for 3 min, followed by 37 cycles each consisting of three steps: 30 s at 94°C (denaturation), 30 s at 58°C (annealing) and 90 s at 72°C (extension). After the last cycle, the extension step was continued for a further 10 min, and then the reaction was held at 4°C.

The nested amplification was carried out in a second tube, with the reaction mix again totaling 20 μl and containing the same reagents as the first stage, except that the primers were now 1 μM primer ITS1F and 1 μM primer ITS2R4, and the target DNA was provided by adding 1 μl of the completed first-stage PCR reaction. Cross-contamination was monitored by negative controls for sample extraction and PCR solutions. The thermocycler program was as described for the first-stage.

The final PCR products of 462 bp were directly sequenced to identify *Leishmania* species infecting individual sand flies. They were purified using the Exonuclease I/Shrimp Alkaline Phosphatase (GE Healthcare, US) before sequencing by using a BigDye Terminator version 3.1 Cycle Sequencing kit (Applied Biosystems, Foster City, CA, USA) and an ABI PRISM 3130 DNA automated sequencer (Applied Biosystems). Sequencing data were analyzed using SeqScape v.2.5 software (Applied Biosystems). Sequences were aligned using the multiple alignment program MEGA 6. A phylogenetic tree was constructed by using the Neighbor-Joining method in agreement with the Kimura 2-parameter model with uniform rates for transitions and transversions. Bootstrap replicates were performed to estimate node reliability, and values were obtained from 1,000 randomly selected samples of the aligned sequence data. Sequences were compared with entries retrieved from GenBank.

### *Leishmania* DNA sequencing and Neighbor-Joining analysis

The sequences obtained (KJ567476, KJ567477, KJ567478, KJ567479, KJ567480, KJ567481, KJ567482) were initially analyzed by the BLAST program; 3 *P. longicuspis* and 4 *P. sergenti* pools were found to be infected by *L. infantum* and *L. tropica* respectively (Table 1). The sequencing results were processed using MEGA software version 6 [24] and the neighbor joining (NJ) method [25] was used to construct the phylogenetic tree. The percentage of replicate trees in which the associated taxa clustered together in the bootstrap test (1000 replicates) are shown next to the branches [26]. All nucleotide sequences obtained in this study were compared using the program ClustalX with sequences from *L. infantum* and *L. tropica* retrieved from GenBank.

**Table 1:** Sand flies collected in the Louata locality: species diversity, relative abundance and infection rates (%)

**Species**	**Male**	**Female**	**Total**	**%**	**Infected sand flies (%)**	***Leishmania *** **species**
*P. longicuspis*	764	1126	1890	71.32	3 (0.26)	***L. infantum***
*P. sergenti*	281	276	557	21.01	4 (1.44)	***L. tropica***
*P. perniciosus*	120	15	135	5.09	-	**-**
*P. papatasi*	18	18	36	1.35	-	**-**
*P. langeroni*	10	10	20	0.75	-	**-**
*P. ariasi*	1	4	5	0.18	-	**-**
*P. chabaudi*	0	1	1	0.03	-	**-**
*S. antennata*	2	0	2	0.07	-	**-**
*S. minuta*	0	3	3	0.11	-	**-**
*S. drefussi*	0	1	1	0.03	-	**-**
**Total**	1196	1454	2650	100	7	**-**

### Blood meal identification

Genomic DNA was extracted from 10 fully engorged sand flies (6 *P. longicuspis* and 4 *P. sergenti*) using the QiaAmp blood DNA mini Kit (Qiagen, Hilden, Germany, Cat. no. 51106), as per the manufacturer’s instructions. The DNA was eluted in 0.1 mL of AE buffer (supplied with the Qiagen kit) after extraction.

DNA of hosts was amplified using PCR with universal primers complementary to the conserved region of mitochondrial DNA (mt DNA) *Cyt b* gene. The primers, L14841 (F5’ CCATCCAACATCTCAGCATGATGAAA 3’) and H15149 (R5’ CCCCTCAGAATGATATTTGTCCTCA 3’) amplified 359 bp of the *Cyt b* gene [27].

A mixture of 25 μL solution containing 1×Taq polymerase buffer B (Promega), 3 mM MgCl_2_, 0.3 mM each dNTP, 0.4 μM primer L14841, 0.4 μM primer H15149, 1 unit Taq polymerase (Promega) with conditions of pre-heating at 95°C for 5 min, 36 cycles of consecutive incubations at 95°C for 30 sec, 60°C for 30 sec, 72°C for 40 sec and 72°C for 5 min. A negative control was included for each batch of assay. Amplified DNA products were confirmed with 1.2% agarose gel and visualized under ultraviolet (UV) light after staining with 2 mg/mL ethidium bromide. A 100 bp DNA ladder was used as the standard marker for comparison.

The final PCR products of 359 bp were purified using the Exonuclease I/Shrimp Alkaline Phosphatase (GE Healthcare, US) before sequencing by using BigDye Terminator version 3.1 Cycle Sequencing kit (Applied Biosystems, Foster City, CA, USA) and an ABI PRISM 3130 DNA automated sequencer (Applied Biosystems). Sequencing data were analyzed using SeqScape v.2.5 software (Applied Biosystems).

To identify vertebrate host species, the obtained unknown sequences were compared with those already deposited in the GenBank database using BLAST program searches (Basic Local Alignment Search Tool, NCBI. Available online from: http://blast.ncbi.nlm.nih.gov). Sequences of a given pair-wise alignment with the lowest E-value were selected as the most likely species of host.

## Results

### Sand fly species diversity and relative abundance

A total of 2650 sand flies (1454 females and 1196 males) were collected in this survey from the Louata locality from June to August 2008. Morphological analysis identified 7 *Phlebotomus* species of three subgenera as follows: *P. longicuspis, P. perniciosus, P. langeroni* and *P. ariasi* of the subgenus *Larroussius; P. sergenti* and *P. chabaudi* of the subgenus *Paraphlebotomus; P. papatasi* of the subgenus *Phlebotomus.* Three *Sergentomyia* species were also identified: *Sergentomyia antennata*, *S. minuta* and *S. drefussi.*

The predominant species in this focus was *P. longicuspis* followed in descending order by *P. sergenti*, *P. perniciosus*, *P. papatasi*, *P. langeroni*, *P. ariasi*, *S. antennata*, *S. minuta*, *P. chabaudi* and *S. drefussi* (Table 1). In June and August, only 5 *Phlebotomus* species were collected; whereas, the highest phlebotomine density and diversity was observed in July; all sand fly species were represented, apart from *P. langeroni* (Table 2).

**Table 2:** Species and abundance of sand flies collected from June to August in the Louata locality

**Species**	**June**	**July**	**August**	**Total**
*P. longicuspis*	444	1186	260	1890
*P. sergenti*	174	369	14	557
*P. perniciosus*	49	68	18	135
*P. papatasi*	10	24	2	36
*P. langeroni*	0	0	20	20
*P. ariasi*	1	4	0	5
*P. chabaudi*	0	1	0	1
*S. antennata*	0	2	0	2
*S. minuta*	0	3	0	3
**Total**	678	1658	314	2650

### *Leishmania* infections in sand flies

Fifty five monospecific pools of female sand flies were screened for *Leishmania* infection by nested PCR. To identify *Leishmania* spp., PCR products were directly sequenced using the nested forward primer ITS1F overlapping the 3’ end of the SSU rRNA gene and ITS1 with the nested reverse primer ITS2R4 at the 5’ end of ITS2. Sequences showed a length range of 390–392 base pairs. Seven pools, 3 *P. longicuspis* and 4 *P. sergenti* were found to be infected by *L. infantum* and *L. tropica*. The seven positive pools were collected from domestic animal shelters and inside houses.

### *Leishmania* DNA sequencing and Neighbor-Joining analysis

Phylogenetic analysis based on the partial sequence of the ITS-5.8S rDNA using the BLAST program showed that the sequences KJ567478 and KJ567477 were similar to the Iranian *L. tropica* sequence deposited in GenBank under the accession number JX560482 (99%), while KJ567476 and KJ567479 were similar to the Moroccan *L. tropica* sequence deposited in GenBank under the accession number KC145155 (99% and 97% respectively).

**Figure 2:**
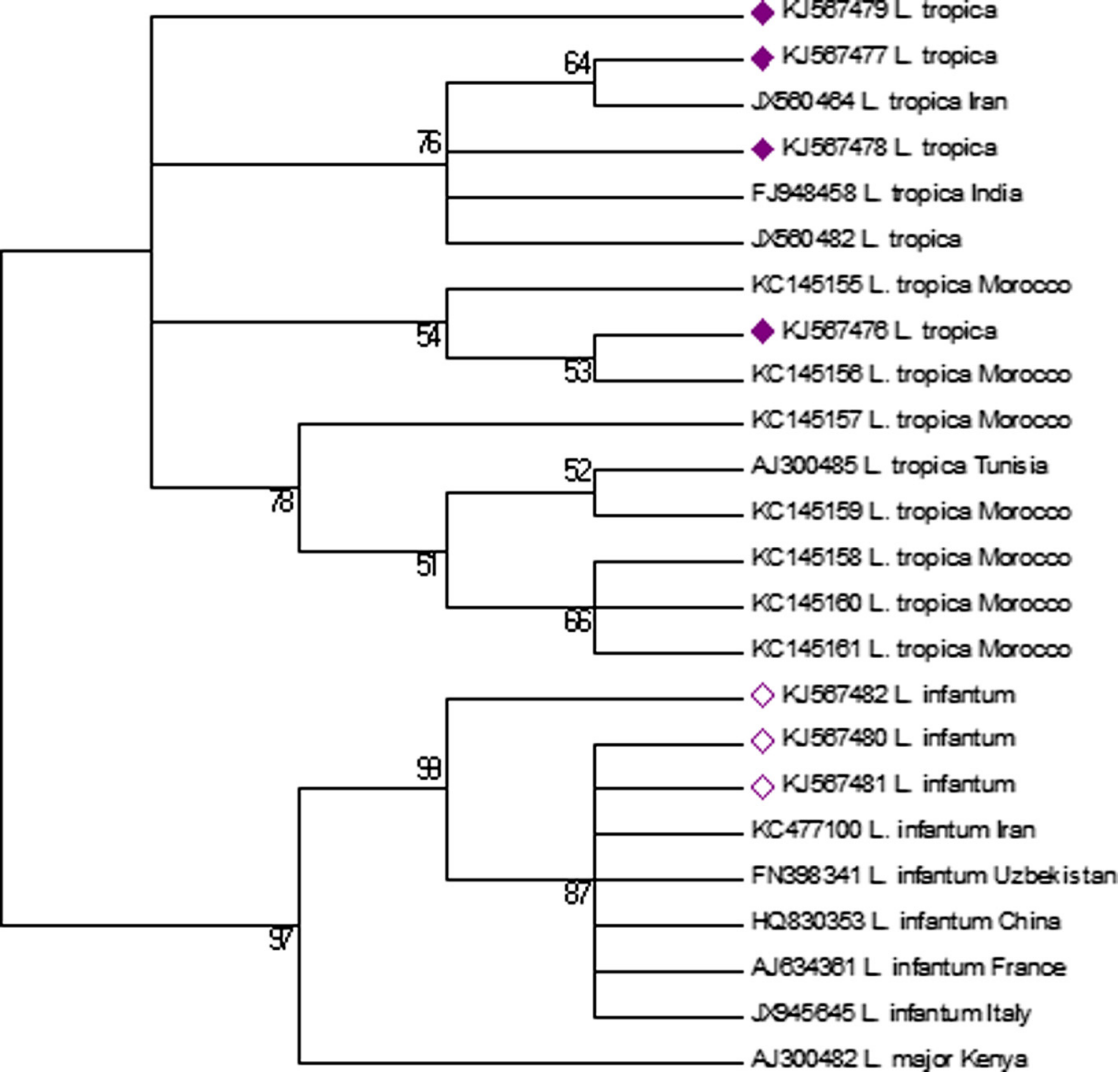
**Neighbor-Joining tree showing the relationships of the sequences of *L. tropica *and *L. infantum *using MEGA 6 software, related to sequences in GenBank.** The evolutionary history was inferred using the Neighbor-Joining method. The optimal tree with the sum of branch length = 0.17897865 is shown. The percentage of replicate trees in which the associated taxa clustered together in the bootstrap test (1000 replicates) is shown next to the branches. The evolutionary distances were computed using the Maximum Composite Likelihood method and are in the units of the number of base substitutions per site. The analysis involved 24 nucleotide sequences. All positions containing gaps and missing data were eliminated. There were a total of 362 positions in the final dataset. Evolutionary analyses were conducted in MEGA6.

On the other hand, the sequences KJ567482 and KJ567481 were similar to the Iranian *L. infantum* sequence deposited in GenBank under the accession number KC477100 (99% and 96% respectively), while KJ567480 was similar to the Italian *L. infantum* sequence deposited in GenBank under the accession number JX945645 (98%) (Figure 2).

### Sand fly blood meal identification

Ten freshly fed female sand flies (6 *P. longicuspis* and 4 *P. sergenti*) collected inside houses and in domestic animal shelters were tested by *Cyt b* PCR to determine the blood meal origin. A positive PCR was obtained for all sand flies tested. Negative control yielded no PCR product implying that only host DNA patterns were detected. Direct sequencing of the 10 amplified parts of the *Cyt b* gene was performed and sequence analysis demonstrated that all sequences were 95% identical to human sequences.

## Discussion

Phlebotomine sand flies are present in periurban, rural and sylvatic environments and distributed in all countries around the Mediterranean basin. Therefore, human populations and domestic animals living in these areas are potential targets of sand fly-borne diseases, such as leishmaniasis. Knowledge of the distribution of the sand fly population is important in predicting the spatial and temporal variations in the risk of leishmaniasis.

In this work, we report the distribution, *Leishmania* infection rate and blood feeding preferences of phlebotomine sand flies found in the Louata locality, where sporadic human cases of CL continue to occur every year. Among 23 species described in Morocco, ten sand fly species (43%) were identified in this area. July had the highest sand -fly abundance and diversity; *P. longicuspis* was clearly the dominant species, exceeding the cumulative abundance of the other species identified (71.32%); this species has been incriminated elsewhere in the transmission of *L. infantum*[28]. The second predominant species identified in this area was *P. sergenti* (21.01%), the confirmed vector of *L. tropica* throughout North Africa, the Middle East and Central Asia [9,29-32].

*P. sergenti* the main vector of *L. tropica* has a wide distribution from sub-Saharan Sahel to the center of Asia through the Middle East and India and is also the most adapted to the Mediterranean climate especially for semi-arid habitats [29].

In Morocco, *L. tropica* was first isolated from *P. sergenti* over three decades ago [30] and recently from *P. sergenti* in support of its vector status, in an emerging CL focus in the Centre of Morocco [3]. In this study, *L. tropica* DNA was found in four *P. sergenti* females; the prevalence of infection was almost 1.44%; this rate may be a consequence of a high level of circulation of this *Leishmania* species in this focus, where cutaneous leishmaniasis is reported to be due to *L. tropica*[12]. Furthermore, the analysis of the blood meal in the *P. sergenti* females showed that they all fed on humans. The relative abundance of *P. sergenti*, coupled to its anthropophilic character, and its high natural infection rate with *L. tropica* among unengorged females, constitutes a strong indication that this species plays a major role as the principal, if not the only vector of *L. tropica* in this area. On the other hand, the screening for *L. tropica* infection of the other sand fly species separated into monospecific pools showed no positive PCR, suggesting that *P. sergenti* is the only vector of *L. tropica* in this area.

*P. longicuspis* has been most frequently recorded in the semiarid, arid and periarid Mediterranean bioclimate zones of north-west Africa [2]. In Morocco, Rioux *et al.* found *P. longicuspis* in all areas, from the sub humid belt to the Sahara, and at varying altitudes [2].

*P. longicuspis* has been considered an important vector of *L. infantum* Nicolle in Algeria since the 1940s [33]. In 1990, Rioux and Lanotte proved its role in the transmission of *L. infantum* in a cutaneous leishmaniasis focus [28]. Recently in Algeria, *L. infantum* DNA was detected in *P. longicuspis* from a visceral leishmaniasis endemic focus [34]. In Morocco, several epidemiological and entomological findings, including the abundance and endophily of this species suggest the capacity of *P. longicuspis* to be a vector; Dereure *et al*. reported that in the pre-Saharan area, *P. longicuspis* is the only species of the subgenus *Larroussius* sufficiently abundant to be suspected of transmitting visceral leishmaniasis [35]. In the same way, and based on its abundance, this species is considered to be a suspected vector of *L. infantum* in a focus of leishmaniasis in northern Morocco [11].

In the present work, *P. longicuspis* was the most predominant species (71.32%), and we report for the first time, both in this focus and in the country, the presence of *L. infantum* DNA within three unengorged females; the results of blood meal analyses by the *Cyt b* gene indicate that *P. longicuspis* is strongly anthroponotic; since all engorged females contained human blood. So, based on its abundance and anthropophily, in addition to the fact that it was the only species found naturally infected with DNA of *L. infantum*, we conclude that *P. longicuspis* can be incriminated as the vector of *L. infantum* in this leishmaniasis focus. This phlebotomine species has never been involved in the transmission of *L. infantum* in Morocco, even if it is a known proven vector elsewhere.

In this work, we applied molecular techniques for the detection of *L. tropica* and *L. infantum* in *P. sergenti* and *P. longicuspis* respectively, as well as in the study of their host preferences. In recent years, molecular techniques have been used increasingly to identify *Leishmania* infection in phlebotomine sand flies, as a sensitive and effective tool useful in epidemiological studies [16,18,36,37]. The increasing application of molecular techniques in this field has considerably reduced the time needed to obtain results. On the other hand, the use of field collected samples without parasite cultivation is interesting in more ways than one, indeed, previous publications showed evidence that cultivation selects subpopulations of the parasites in the biological samples, including co-existence of non-pathogenic trypanosomatids [38]. In addition, direct analysis of *Leishmania* DNA in field collected samples produced an unexpected outcome, suggestive of *L. tropica* or *L. infantum* or a mixture of the two in CL lesions [39]. PCRs based on the amplification of the ITS-5.8S rRNA gene have been used for molecular detection of *Leishmania* spp. in biological samples and have proved to be highly sensitive [22]. Indeed, this PCR approach has been applied successfully in the detection of *Leishmania* spp. in entomological surveys [3,22]. In addition, we used the *Cyt b* to identify blood meals, which has been widely employed due to its high copy numbers and sufficient genetic variation among vertebrate taxa at the primary sequence level [40-42].

Our results highlight two findings. Firstly, *P. longicuspis* was more abundant than *P. sergenti* in this cutaneous leishmaniasis focus, where, even if *L. tropica* was identified in CL [12], it may not be the only species responsible for the disease, as the survey was done only on a small number of skin samples [12]. Secondly, we report for the first time the natural infection of *P. longicuspis* with *L. infantum* in Morocco, which incriminates it as a vector of this parasite. The presence of *L. infantum*, and its presumed vector in this area, where cutaneous leishmaniasis is already widespread, makes this a site of high risk of visceral leishmaniasis, mostly because of the proximity of a human and canine visceral leishmaniasis focus, less than 20 km from this investigated area [43]. Furthermore, the absence of VL cases does not mean that this locality is VL free, since most *L. infantum* visceral infections remain asymptomatic. More epidemiological investigations are needed to demonstrate the role of *L. infantum* circulating in this area in the transmission of visceral and/or cutaneous leishmaniasis.

## Conclusion

This focus of leishmaniasis, where *L. tropica* and *L. infantum* co-exist appears peculiar. This result has important implications for drawing attention to the diagnosis and treatment of *Leishmania* infections. Programs and/or monitoring projects should be developed to detect risk of exposure to mixed infections with *Leishmania spp.* The follow up of patients with cutaneous leishmaniasis living in this area is necessary in order to reach a better understanding of the interactions between the two parasites.

## Competing interests

Authors declare that they have no competing interests.

## Authors’ contributions

NE Carried out the laboratory work of entomological and molecular studies, analyzed data, and prepared the manuscript. MA Contributed to the entomological studies and data analysis. ALI is involved in the study design. MF is involved in the review of the manuscript. ML designed the study, entomological studies, contributed to interpretation, analyzed and finalized the manuscript. All the authors read and approved the final version of the manuscript.
